# Green Solutions for Food Safety: The Emerging Applications of Zearalenone-Degrading Enzymes

**DOI:** 10.3390/foods14173010

**Published:** 2025-08-28

**Authors:** Yawei Zhang, Xianfeng Ren, Baocheng Xu, Lixia Fan, Changying Guo, Bingchun Zhang, Mingxiao Ning

**Affiliations:** 1 Laboratory of Quality and Safety Risk Assessment for Agro-Products of the Ministry of Agriculture (Jinan), Institute of Quality Standard and Testing Technology for Agro-Products, Shandong Academy of Agricultural Sciences, Jinan 250100, China; m13792135609@163.com (Y.Z.); renxianfenga@163.com (X.R.); superdemeter@163.com (L.F.); cyguo808@163.com (C.G.); llzbest66@163.com (B.Z.); 2 International Joint Laboratory of Food Green Processing and Safety Control, College of Food and Bioengineering, Henan University of Science and Technology, Luoyang 471000, China; xbc76@163.com

**Keywords:** zearalenone, lactone hydrolase, molecular modification, biodegradation

## Abstract

Zearalenone (ZEN), a mycotoxin produced by *Fusarium* species, widely contaminates grains and feed, posing a serious threat to animal and human health. Traditional physical and chemical detoxification methods face challenges, including low efficiency, high costs, and nutrient loss. In contrast, enzymatic biodegradation has emerged as a research hotspot due to its high efficiency, specificity, and environmental friendliness. Lactone hydrolase can specifically hydrolyze the lactone ring of ZEN, converting it into a low-toxicity or non-toxic degradation product, thereby demonstrating significant potential for application in ensuring the safety of food, feed, and agricultural products. In recent years, with advancements in enzyme engineering and various biological technologies, remarkable progress has been made in ZEN-degrading enzyme research. Novel and highly efficient enzyme genes have been discovered through gene mining, while directed evolution and rational design have improved catalytic efficiency and stability. Additionally, immobilization techniques and formulation optimization have enhanced industrial applicability. This review, based on practical application needs, establishes a comprehensive evaluation system integrating enzyme characteristics, modification technologies, and process applicability, aiming to provide actionable theoretical guidance for the large-scale application of biological detoxification technologies.

## 1. Introduction

Zearalenone (ZEN), also known as F-2 toxin, is a mycotoxin primarily produced by various *Fusarium* species, including *Fusarium graminearum*, *Fusarium roseum*, *Fusarium moniliforme*, *Fusarium culmorum* and *Fusarium tricinctum*. It was first isolated and identified in 1962 from moldy corn inoculated with *F. graminearum* [1]. ZEN contamination is widespread in cereal crops, with corn, wheat, and oats being the most susceptible to contamination [2]. Studies have shown that ZEN is a dihydroxybenzoic acid lactone compound with five structural derivatives: α/β-zearalenol (α/β-ZEL), α/β-zearalanol (α/β-ZAL), and zearalanone (ZAN) [3]. The chemical structure of ZEN and its derivatives contains a phenolic dihydroxy lactone moiety that structurally resembles natural estrogens. Upon entering human or animal bodies, they competitively bind to estrogen receptors, exhibiting estrogen-like activity. This interaction disrupts the endocrine system, leading to various toxic effects, including reproductive toxicity, genotoxicity, carcinogenicity, and immunotoxicity [4]. Consequently, consumption of ZEN-contaminated food or feed poses serious risks to human and animal health and causes significant economic losses.

Given these concerns, rapid and effective methods for ZEN degradation and detoxification have attracted increasing research attention. Currently, ZEN detoxification approaches mainly include physical, chemical, and biological methods. Physical detoxification methods demonstrate significant advantages in zearalenone (ZEN) detoxification, including operational simplicity, cost-effectiveness, and suitability for large-scale processing [5]. The primary approaches include adsorption, irradiation, and milling methods. The adsorption method enables rapid toxin concentration reduction through readily available adsorbents (e.g., activated carbon and bentonite); however, its non-selective binding properties may concurrently deplete essential nutrients, thereby compromising product quality [6]. Irradiation techniques effectively disrupt the molecular structure of toxins without requiring chemical additives yet may alter food nutritional composition and cause economic losses [7,8]. The milling process physically removes contaminated portions but shows limited efficacy and may damage grain nutrients [9]. Chemical detoxification methods such as oxidant, alkali treatment or ozone can show rapid reaction kinetics and show special effectiveness in treating highly polluted materials. However, these methods are accompanied by many disadvantages, including complicated processing requirements, destruction of nutrients, reduced palatability and potential harm of chemical residues [10,11,12].

Biodegradation primarily employs two distinct strategies: microbial-based biotransformation and enzymatic detoxification. The former utilizes bacterial or fungal strains for toxin degradation, offering operational simplicity and cost-effectiveness, yet its industrial application in food and feed sectors is constrained by inherent limitations including biosafety risks, intricate metabolic pathways, and suboptimal growth kinetics [13]. In contrast, enzymatic detoxification leverages highly specific and catalytically efficient enzyme systems to achieve precise toxin removal, exhibiting superior technical merits such as mild processing conditions, stringent substrate specificity, and absence of secondary pollutants, thereby representing the most promising approach for future food safety interventions [14,15]. Current research has identified three major classes of enzymes capable of degrading ZEN: peroxidases, laccases, and lactone hydrolases. Peroxidases are widely present in animals, plants, and microorganisms. They primarily achieve detoxification by oxidatively cleaving the dihydroxyphenyl ring structure in ZEN molecules and have been successfully expressed in *E. coli*, *Pichia pastoris*, and *Saccharomyces cerevisiae*. However, the large-scale application of peroxidases in food and feed processing is impractical due to low market acceptance, leaving their practical value pending further development [16,17,18,19]. Laccase was first discovered in the secretions of the Japanese lacquer tree and was later confirmed to be widely distributed in plants, bacteria, insects and fungi [20]. Laccases, as multicopper oxidases, primarily act on ZEN’s biphenol structure. However, these enzymes exhibit relatively low catalytic activity toward ZEN and often require mediators to enhance degradation efficiency, making them more suitable for aflatoxin degradation [21,22,23]. In comparison, lactonase hydrolases demonstrate remarkable advantages in ZEN detoxification due to its cofactor-independent catalytic properties and high degradation efficiency, exhibiting significant research value and broad application potential [24].

We systematically searched Google Scholar, CNKI, and Web of Science databases using the keywords zearalenone, zearalenone-degrading enzymes, lactone hydrolase, and immobilization applications to comprehensively compile relevant reviews and research articles published between 1962 and 2025. This article systematically reviews the characteristics of ZEN lactonase, molecular modification strategies, and practical application progress, aiming to provide theoretical references for the development and industrial application of this type of detoxification enzyme.

## 2. Enzymatic Characteristics of Lactone Hydrolase

### 2.1. Structural Characteristics and Catalytic Properties of Lactone Hydrolases

Lactonases play a crucial role in the degradation of ZEN, a process of significant importance in various biological and industrial applications. These enzymes possess unique structural features, such as specific active site architectures and catalytic motifs, which confer remarkable substrate specificity and catalytic efficiency [25]. This section will explore the key structural characteristics and catalytic mechanisms of lactonases, providing a theoretical foundation for subsequent enzyme engineering and industrial applications.

#### 2.1.1. Structural Characteristics of Lactone Hydrolases

**Figure 1:**
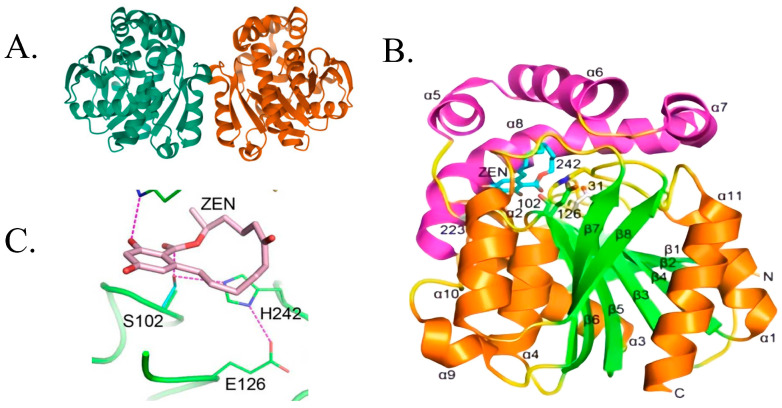
The structure diagram of ZHD101. Note: (**A**). Overall structure diagram of ZHD101 (PDB codes: 3WZL). https://doi.org/10.2210/pdb3WZL/pdb, accessed on 23 June 2025. (**B**). Three-dimensional spatial diagram of ZHD101 combined with ZEN (PDB codes: 3WZM). The central β-sheet and flanking α-helices within the core domain are colored green and orange, respectively. The α-helices of the cap domain are shown in magenta. Side chains of Ser102, His242, Glu126, Asp31, and Asp223, along with the bound substrate from the S102A/ZEN complex, are represented as stick models. (**C**). Catalytic triad of ZHD101. Reprinted (**B**,**C**) with permission from Ref. [26]. 2025, Peng et al.

In 2014, Peng et al. [26] successfully determined the three-dimensional structure of zearalenone hydrolase (ZHD) for the first time using X-ray crystallography, marking a milestone discovery in the structural characterization of this enzyme family (Figure 1). The study revealed that the enzyme exists as a homodimer stabilized without disulfide bonds. Each monomer consists of two characteristic domains: a catalytic domain and a cap domain. The catalytic domain exhibits the typical α/β hydrolase fold, with its core framework composed of eight parallel β-strands (β1–β8) flanked by seven α-helices (α1–α4 and α9–α11). The cap domain is located at the C-terminal edge of the β-sheets and consists of four α-helices (α5–α8). Notably, the α8 helix adopts a unique bent conformation spanning the top of the catalytic domain, a distinctive structural feature critical for substrate recognition. The enzyme’s active center comprises the classic catalytic triad (Ser102-His242-Glu126) [27]. Structural analysis showed that ZEN binds in a deep pocket formed between the catalytic and cap domains in a bent conformation, with its benzoate ring forming a T-shaped π-π stacking interaction with the indole ring of Trp183, while the NE1 of Trp183 forms a hydrogen bond with the ortho-hydroxyl group of ZEN. Additionally, hydrophobic residues such as Leu33 and Val153 interact with the substrate through van der Waals forces, collectively forming a highly specific binding pocket. This explains the remarkable substrate specificity exhibited by ZHD in degrading ZEN [26,28].

In recent years, with deepening research, crystal structures of homologous enzymes from different species have been successively resolved. For example, ZHDAY3 from *E.aquamarina CBS 119918* [29] also possesses the typical S102-H242-G126 catalytic triad, with the histidine residue positioned 3.6 Å and 3.1 Å from the serine and glutamate residues, respectively. Compared to ZHD101 (*Clonostachys rosea IFO 7063*) [30], the most significant structural difference in ZHDAY3 lies in the η5 and α4 regions (residues 132–169) of the cap domain, which exhibit a more pronounced inward depression toward the substrate-binding pocket. Notably, residues in this region not only participate in the construction of the active pocket but also play a crucial role in enzyme-substrate recognition, which may be the key structural basis for the differences in substrate selectivity between ZHDAY3 and ZHD101. Lin et al. [31] modified the electrostatic environment of His242 by selecting surface residues D157 and E171 as mutation sites, replacing the original negatively charged residues with positively charged lysine (D157K and E171K). This modification lowered the enzyme’s pKa value and resulted in significantly improved catalytic efficiency, with kcat/KM values reaching 1.34-fold and 2.06-fold that of the wild-type, respectively. Based on the above analysis, the structural features of enzymes enable precise regulation of their catalytic functions. The geometric configuration of the active center determines the specificity of substrate binding, while the three-dimensional arrangement of key catalytic residues establishes an efficient catalytic environment (including optimal temperature and pH conditions). Furthermore, dynamic conformational changes in the protein may further optimize the stabilization of the reaction transition state. The synergistic interplay of these factors ultimately achieves both high efficiency and substrate selectivity in enzymatic reactions.

#### 2.1.2. Catalytic Mechanism

**Figure 2:**
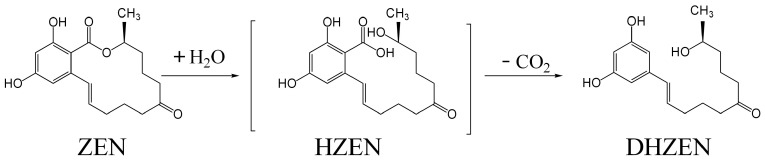
Degradation pathway of ZEN. Note: Adapted with permission from Ref. [30]. 2025, Takahashi-Ando et al.

The catalytic mechanism of lactone hydrolase primarily relies on the specific hydrolysis of the lactone ring. This process involves the nucleophilic attack by a serine residue (Ser-OH) in the enzyme’s active center on the carbonyl carbon of ZEN’s lactone ring, cleaving the ester bond and forming an acyl-enzyme covalent intermediate while simultaneously opening the lactone ring. Subsequently, a water molecule, assisted by histidine, attacks this intermediate to complete the hydrolysis, yielding hydrolyzed zearalenone (HZEN). The hydrolyzed product then spontaneously decarboxylates into non-toxic decarboxylated hydrolyzed zearalenone (DHZEN), which cannot bind to estrogen receptors, thereby achieving detoxification [30,32] (Figure 2). Qi et al. [32] successfully captured the fine conformation of the ZHD-product complex through high-resolution (1.60 Å) crystal structure determination. Structural analysis revealed that after lactone bond cleavage, the phenolic ring region undergoes significant displacement, moving closer to residues Leu132, Tyr187, and Pro188, while the lactone ring region remains relatively stationary. By comparing the structural differences between ZHD-substrate and ZHD-product complexes, it was found that the interaction site of Trp183 N”1 shifts from O2 to O12′. This critical change confirms the important role of Trp183 in driving the unidirectional translational movement of the phenolic ring, providing decisive evidence for elucidating the catalytic mechanism of zearalenone hydrolysis.

Fruhauf et al. [2] systematically assessed the estrogenic potential of HZEN and DHZEN, metabolic products generated through Zhd101 esterase-mediated ZEN hydrolysis, using in vitro evaluation systems. Their quantitative analysis revealed that both metabolites displayed significantly reduced estrogenic activity, showing at least 50–10,000-fold lower potency than the parent ZEN compound under in vitro conditions. In a related study, Christiane Gruber-Dorninger et al. [33] investigated the application of hydrolase ZENA as a feed additive. Their findings revealed that this enzymatic supplement not only catalyzed the degradation of ZEN into non-estrogenic metabolites (HZEN and DHZEN) but also effectively inhibited α-ZEL formation in bovine ruminal fluid. In subsequent research, the same team evaluated ZENA’s efficacy in the gastrointestinal tracts of three monogastric species (*Sus scrofa*, *Gallus gallus*, and *Oncorhynchus mykiss*). The enzyme demonstrated high degradation efficiency against ZEN while concurrently suppressing the production of highly toxic metabolites in these animal models [34].

### 2.2. Sources of Lactone Hydrolase, Catalytic Activity and Reaction Conditions

Lactonase widely exists in microorganisms, plants, and animals, but the lactone hydrolase used to degrade zearalene at present mainly comes from microorganisms. In 2002, Takahashi-Ando et al. [30] first isolated a lactone hydrolase capable of degrading ZEN from *Clonostachys rosea* IFO 7063, designating it as ZHD101. Their study revealed that ZHD belongs to the α/β hydrolase family and demonstrated ZEN-degrading activity in heterologous hosts, including the fission yeast *Schizosaccharomyces pombe* and *Escherichia coli* carrying the cloned gene. In addition, for industrial applications, researchers have also developed a variety of lactone hydrolases from microorganisms (Table 1), such as ZHD607 [35] from *Phialophora americana*, ZENC [36] from *Neurospora crassa*, ZHDAY3 [29] from *Exophiala aquamarina CBS119918*, and ZENM [37] from *Monosporascus* sp. *GIB2*.

The catalytic activity of lactone hydrolase shows strong dependence on both pH and temperature of the reaction system, which are critical determinants of enzymatic efficiency and industrial applicability [56]. Current studies indicate that most reported lactone hydrolases exhibit optimal ZEN-degrading activity under weakly alkaline conditions (pH 7.0–10.0) (Table 1). However, this pH preference substantially differs from physiological conditions in animal gastrointestinal tracts: the stomach maintains a strongly acidic environment (pH 2.0–4.0), while intestinal pH ranges from weakly acidic to neutral (pH 5.0–7.0). Should lactone hydrolase undergo rapid inactivation under acidic conditions, its ZEN-degradation capacity would be severely compromised [57]. Furthermore, temperature represents another crucial factor affecting enzymatic performance. An ideal lactone hydrolase should maintain high catalytic activity within the mesophilic temperature range (25–45 °C) to accommodate both animal physiological temperatures and feed processing/storage requirements [58].

The originally isolated ZHD101 enzyme from *Clonostachys rosea* exhibits optimal activity under alkaline conditions (pH 9.0–10.0), but its activity dramatically decreases to only 34% of initial levels at acidic pH (5.5) [30]. Furthermore, this enzyme demonstrates poor thermal stability, with an optimal temperature range of 37–45 °C, and complete inactivation occurs after just 6 min at 50 °C. In contrast, ZENH from *Aeromicrobium* strain [40] shows peak activity at pH 7.0 and 55 °C, maintaining 7-fold higher activity than ZENG at pH 5.0 (retaining 33% activity) and preserving over 80% of maximal activity at 60 °C, although it cannot be classified as a thermostable enzyme. Similarly, RmZHD from *Rhinocladiella mackenziei CBS* [48] achieves maximum activity at pH 8.6 and 45 °C, but retains merely 8.3% of peak activity when the temperature rises to 55 °C. As summarized in Table 1, current lactone hydrolases show significant mismatch with the acidic tolerance required for feed industry applications, while their thermal stability also requires substantial improvement. Therefore, developing novel lactone hydrolases with combined acid resistance and thermostability is crucial for practical applications, which would significantly enhance their degradation efficiency and application potential in animal feed.

## 3. Molecular Modification and Optimization of Lactone Hydrolase

Molecular engineering and optimization of lactone hydrolase primarily employ directed evolution, rational design, and computer-aided strategies to precisely modulate enzymatic properties, including acid tolerance, stability, catalytic efficiency, and substrate specificity. Directed evolution, independent of precise structural knowledge, generates diverse mutant libraries via random mutagenesis or targeted recombination techniques such as saturation mutagenesis (SM), site-saturation mutagenesis (SSM), error-prone PCR (epPCR), and DNA shuffling. Coupled with high-throughput screening, this approach can serendipitously yield optimized variants. However, its stochastic nature necessitates extensive screening and may introduce deleterious mutations, resulting in reduced efficiency. In essence, directed evolution is particularly suitable for engineering enzymes with undefined mechanisms or complex traits, albeit with higher costs and longer time requirements [59,60]. Rational design leverages established structure-function relationships to implement site-specific mutations or structural modifications of key amino acid residues, thereby precisely modulating catalytic activity, stability, or selectivity. This strategy enables accurate targeting of critical residues with high efficiency and minimal mutant generation. Nevertheless, it demands comprehensive structural insights and mechanistic understanding, while remaining limited in predicting long-range mutations or multi-site synergistic effects. This method proves most effective for well-characterized enzyme systems but exhibits constrained capability for optimizing complex traits [61]. Computer-aided strategies extend rational design by employing computational simulations and structural analyses to predict functional hotspots, followed by precision engineering through site-directed mutagenesis to enhance various enzyme properties. While this approach reduces experimental workload by leveraging high-resolution structural data and molecular modeling technologies, it similarly requires thorough mechanistic comprehension of the target enzyme [62]. Table 2 shows the specific optimization methods and their effects. Each of these strategies has distinct advantages, and their combined application in industrial settings often yields synergistic effects.

### 3.1. Directed Evolution Technology Strategy

Directed evolution is an approach employed when the structural and catalytic mechanisms of an enzyme remain unknown. It utilizes mutagenesis techniques such as error-prone PCR, DNA recombination, and site-saturation mutagenesis to generate sequence-diverse random mutant libraries. These libraries are then subjected to specific selection pressures or conditions to screen for mutants exhibiting desired functional characteristics [58]. Qiu et al. [63] employed a combinatorial mutagenesis approach to engineer the Zhd101 enzyme, ultimately identifying the optimal mutant V153H-V158F (designated as Zhd101.1). The researchers successfully integrated this mutant gene into the food-grade recombinant yeast strain *Kluyveromyces lactis* GG799 (pKLAC1-Zhd101.1), achieving efficient inducible expression and secretion. Systematic enzymatic characterization revealed that the mutant exhibited a 1.1-fold increase in specific activity compared to the wild-type, along with significantly improved thermal stability and pH stability. Dotsenko et al. [64] engineered the catalytic residues E126 and H242 of recombinant ZHD enzyme through site-directed mutagenesis, successfully reducing their pKa values. Notably, the T216K mutant shifted the optimal pH by 1 unit toward acidity, broadened the active pH range, and enhanced activity at pH 3–10. The T216K variant, with superior catalytic efficiency at low pH, proved ideal for ZHD’s decontamination applications. This study not only validated the effectiveness of directed evolution in enzyme functional optimization but also provided critical theoretical foundations for subsequent rational design.

### 3.2. Rationally Design Technical Strategies

Rational and semi-rational design strategies involve targeted enzyme modifications based on a thorough understanding of their sequences, structures, and catalytic mechanisms. Xu et al. [67] conducted a structural analysis of ZHD101 complexes with two ZOL isomers and modified residues surrounding the lactone ring to enhance selective degradation of α-ZOL. The V153H mutant demonstrated a 3.7-fold increase in specific activity toward α-ZOL while maintaining its activity against ZEN. Xing et al. [66] employed a two-step rational design approach to perform multi-site combinatorial mutations on the ZEN-degrading enzyme ZHD101, evolving a variant capable of efficiently hydrolyzing ZEN under acidic conditions. At 37.0 °C and pH 4.2, the evolved enzyme demonstrated a 4.03-fold increase in specific activity and exhibited superior ZEN hydrolysis efficiency in simulated porcine gastric acidic conditions. Zhang et al. [50] successfully purified a recombinant zearalenone-degrading lactonase (ZENG), which, based on available data, is the first reported recombinant enzyme to retain excellent activity and stability at pH 7.0. Through structural and sequence analysis, they designed three double-site mutants H143F/S143F, H143L/S143L, and H143I/S143I to improve ZENG’s thermostability. Among these, the H143F/S143F and H143L/S143L mutants showed significant enhancements in thermal stability compared to the wild-type enzyme. Xue et al. [68] heterologously expressed ZHD11A from Phialophora macrospora and modified it via semi-rational design, obtaining a mutant, I160Y-G242S, which retained approximately 40% residual activity after 10 min at 55 °C. Additionally, the specific activity of I160Y-G242S increased twofold, from 220 U/mg to 450 U/mg, compared to the wild-type ZHD11A. Ouyang et al. [37] conducted site-directed mutagenesis on five conserved residues (L123, G163, E171, S199, and S202) in ZENM based on sequence-structure alignment with other ZEN lactonases. Notably, the G163S mutation within the cap domain demonstrated significantly enhanced catalytic activity toward α-ZOL relative to the wild-type enzyme. Kinetic analysis revealed that this mutant exhibited superior catalytic efficiency (kcat/Km) for α-ZOL compared to ZEN, establishing α-ZOL as both the preferred substrate and a key determinant of substrate specificity for this lactonase.

In summary, rational and semi-rational design strategies have proven highly effective in the engineering of zearalenone-degrading enzymes. These approaches have not only expanded the enzymes’ pH adaptability but also significantly improved their catalytic efficiency, thermostability, and specific activity. These advancements provide powerful enzymatic tools for the biodegradation of zearalenone contamination in food and feed, while also establishing a technical paradigm for the rational engineering of other industrial enzymes—namely, structure- and function-guided precision design combined with computational modeling can systematically optimize enzymatic performance and environmental adaptability. Future integration of AI-driven predictions and high-throughput screening technologies is expected to accelerate the development of enzymes with multi-objective synergistic enhancements.

### 3.3. Computer-Aided Rational Design Strategy

Computer-aided rational design minimizes reliance on trial-and-error experimentation and significantly reduces the workload of mutant screening. By employing computational tools, researchers can predict single-point mutations likely to enhance target enzyme properties. Molecular dynamics simulations then assess the structural and functional impacts of these mutations, enabling the elimination of unfavorable variants prior to experimental validation. Ultimately, validated thermostabilizing mutations are combinatorially integrated to generate highly improved multipoint mutants with significantly enhanced performance [75].

Wang et al. [29] identified and characterized a broad-spectrum zearalenone hydrolase ZHDAY3 derived from *E. aquamarina CBS 119918*. Through rational design, the engineered ZHDAY3 (N153H) mutant exhibited a specific activity of 253.3 ± 4.3 U/mg toward α-ZAL. Integrated approaches, including molecular docking, structural comparison, and kinetic analyses, collectively demonstrated that the enhanced catalytic efficiency primarily resulted from the reduced distance between the His242 side chain (catalytic residue) and the α-ZAL lactone bond, along with improved enzyme-substrate binding affinity. Lin et al. [31] successfully engineered the pH adaptability of ZHD101 using rational design coupled with computational strategies, enabling the enzyme to maintain high catalytic activity under acidic conditions. By altering the electrostatic environment of His242, the researchers selected surface residues D157 and E171 as modification sites, replacing the negatively charged residues with positively charged lysine (D157K and E171K) to lower the enzyme’s pKa. This adjustment prevented protonation in acidic solutions, shifting the pH-activity profile toward the acidic range. The resulting mutants, M2 (D157K) and M9 (E171K), exhibited significantly enhanced catalytic efficiency at pH 5.5, with kcat/KM values reaching 1.34-fold and 2.06-fold that of the wild-type enzyme, respectively. Fang et al. [46] identified two highly flexible residues with elevated root-mean-square fluctuation values through molecular dynamics simulations. Site-directed mutagenesis at these positions yielded the S162P/S220R mutant showing a 36.8-fold increase in enzymatic activity at 55 °C compared to wild-type. Structural analysis revealed that the thermostability improvement stemmed from newly formed hydrogen bonds, salt bridges, and proline substitution. Furthermore, virtual saturation mutagenesis targeting thermolabile regions (e.g., specific segments of ZLHY-6) generated the H134W mutant retaining significantly higher activity after 20 min heat treatment at 45 °C, exhibiting 10-fold greater activity than wild-type. These findings demonstrate that flexibility analysis coupled with virtual mutagenesis effectively enhances lactonase thermostability. Chen et al. [72] employed MD simulations to identify mutation sites followed by virtual saturation mutagenesis. The selected mutants (N156F, S194T, and T259F) improved thermostability through salt bridge rearrangement, NH-π interaction formation, and surface cavity filling. Du et al. [73] utilized Discovery Studio to predict disulfide bond introduction in ZLHY-6, obtaining mutants H134W and Q45C/A253C that maintained 10-fold and 3.1-fold relative activities, respectively, after 45 °C/20 min treatment, while preserving ZEN degradation activity (41.33% and 102.67%). Subsequent MD and structural simulations suggested the mutations likely reduced conformational fluctuations and enhanced structural rigidity, thereby improving protein thermostability.

In summary, current enzyme engineering approaches predominantly rely on structure-based and sequence-based comparative analyses to generate mutants from wild-type enzymes. These are further assisted by molecular dynamics simulations and other modeling techniques to elucidate various enzyme catalytic mechanisms, ultimately yielding an optimized mutant enzyme.

## 4. Application Research of Lactone Hydrolase

Recent research has made significant progress in the application of ZEN detoxification strategies. A prominent approach involves direct biodegradation using microbial-derived enzymes or molecularly engineered free enzymes, which has gained considerable attention due to its high efficiency and environmentally friendly characteristics, as previously described. Furthermore, the implementation of enzyme immobilization techniques, including carrier adsorption, covalent binding, and nanomaterial encapsulation, has substantially enhanced enzyme stability, reusability, and operational efficiency in industrial detoxification processes [76]. These technological advancements not only improve the sustainability of toxin degradation but also offer scalable solutions for food and feed safety applications.

### 4.1. Direct Detoxification Application of Free Enzyme

Free-form ZEN lactonase demonstrates significant potential for direct food detoxification applications due to its high catalytic activity and operational simplicity. Unlike immobilized enzymes, free enzymes can directly interact with contaminated substrates without requiring complex carrier modifications, making them particularly suitable for liquid food systems or products amenable to soaking treatments [77]. However, their environmental sensitivity (to factors such as pH and temperature) and challenges in recovery/reuse significantly limit their large-scale industrial applications.

#### 4.1.1. Detoxification Applications in Food

With increasingly stringent requirements for food safety, the application of ZEN-lactone hydrolase in the detoxification of food products has gradually advanced. This enzyme can efficiently degrade ZEN under mild conditions (e.g., neutral pH, room temperature) without affecting the sensory quality or nutritional value of food. Chang et al. [78] demonstrated that during corn oil refining, after neutralization and enzymatic detoxification with Zlhy-6, the ZEN content in crude oil decreased from 1257.3 μg/kg to 13 μg/kg (a residual rate of 3.69%). No significant differences were observed in the total tocopherol and sterol content after enzymatic detoxification, and degradation products were completely removed through washing. This study provided critical evidence for the industrial application of enzymatic ZEN detoxification in corn oil production. Zhao et al. [79] optimized the process of ZEN removal from degummed corn oil (DCO) using hydrolase in a batch refining system through single-factor and response surface experiments. Under the optimized conditions (temperature 39.01 °C, pH 8.08, time 3.9 h, enzyme dosage 44.7mg/kg), the degradation rate of ZEN reached 94.66%. Under these conditions, the initial ZEN concentration influenced the removal efficiency, with higher ZEN levels leading to faster degradation rates, whereas lower levels resulted in slower degradation. Xiang et al. [53] found that treating wort with ZHD before fermentation removed approximately 90% of ZEN within 12 min of enzyme addition. ZHD remained effective in degrading ZEN after cooling via a plate heat exchanger. However, once fermentation began, the detoxification efficiency of the enzyme significantly declined due to interference from Saccharomyces cerevisiae metabolism and temperature fluctuations. In contrast, Bi et al. [36] reported that treating distillers’ dried grains with solubles (DDGS) required 24 h to achieve a 71% degradation rate, whereas corn byproducts needed 48 h to reach 89% degradation. Corn bran, however, showed a faster response, with 88% degradation achieved in just 3 h and 94.7% in 6 h. Chen et al. [80] applied ZHD101 (crude enzyme solution) to the detoxification of ZEN in corn steep liquor. The detoxification rate increased with prolonged reaction time, with ZEN (approximately 25 μg/mL) degraded by (6.2 ± 1.5%), (13.4 ± 2.8%), and (17.1 ± 8.0%) after 1, 2, and 3 h of reaction, respectively. Wang et al. [81] used *Kluyveromyces marxianus*-fermented recombinant ZHD101 to treat moldy corn, achieving complete ZEN degradation within 12 h.

Studies have demonstrated that ZEN-lactone hydrolase exhibits efficient detoxification capabilities across various food matrices, including corn oil, wort, distillers’ grains, and corn byproducts. Its degradation efficiency is influenced by factors such as temperature, pH, reaction time, and initial ZEN concentration. Under optimized conditions, a degradation rate exceeding 90% can be achieved while effectively preserving the nutritional components of food. These findings provide reliable technical support for industrial-scale applications.

#### 4.1.2. Detoxification Application in Feed or Livestock and Poultry Animals

Wang et al. [82] added ZEN-jjm expressed by genetically modified *Bacillus subtilis* to feed, which could remove high concentrations of ZEN. Evaluation based on indicators such as total number of piglets born, number of stillbirths, number of weak piglets, and piglet birth weight demonstrated that the constructed ZEN-degrading enzyme could effectively degrade ZEN’s toxic effects on sow gestation performance and reproductive performance during pregnancy. Song et al. [83] similarly evaluated the protective effects of a novel ZEN lactonase Zymdetox Z-2000 (derived from *Bacillus subtilis*) on the growth performance and reproductive health of replacement gilts. Results showed that compared with the CON group, the ZEN group exhibited reduced weight gain, increased vulva area and reproductive organ indices, elevated serum AST activity and estradiol (E2) levels, induced ovarian tissue lesions, decreased uterine total antioxidant capacity (T-AOC) while increasing ovarian T-AOC, and increased ZEN residues in the stomach and duodenum. All three additives could alleviate ZEN toxicity, with Zymdetox Z-2000 (especially the coated version) showing significantly better effects than *B. subtilis*. The conclusion confirmed that ZEN damages replacement gilt growth and reproductive functions, while Zymdetox Z-2000 could effectively reduce its reproductive toxicity by degrading ZEN into hydrolyzed products with low estrogenic activity. Christiane Gruber-Dorninger et al. [33] administered a single oral dose of ZEN to non-lactating *Holstein cows* (*n* = 4) with rumen fistulas and measured ZEN and ZEN metabolite concentrations in free rumen fluid from three reticuloendothelial locations (reticulum, ventral sac, and dorsal mat layer) over 34 h. The study showed that adding zearalenone hydrolase ZenA significantly reduced ZEN and its estrogenic metabolite levels in various rumen compartments and converted them into non-estrogenic hydrolyzed product HZEN, indicating ZenA’s potential as a feed additive for alleviating ZEN’s estrogenic toxicity in cattle.

These research results demonstrate that free enzymes can be directly used for ZEN detoxification, but their actual application effects may be significantly constrained by multiple factors such as material characteristics, processing time, and environmental conditions. We still need to further optimize enzyme preparation forms or improve processing technologies to enhance their stability and working efficiency in complex systems.

### 4.2. Application of Immobilized Enzymes for Detoxification

**Figure 3:**
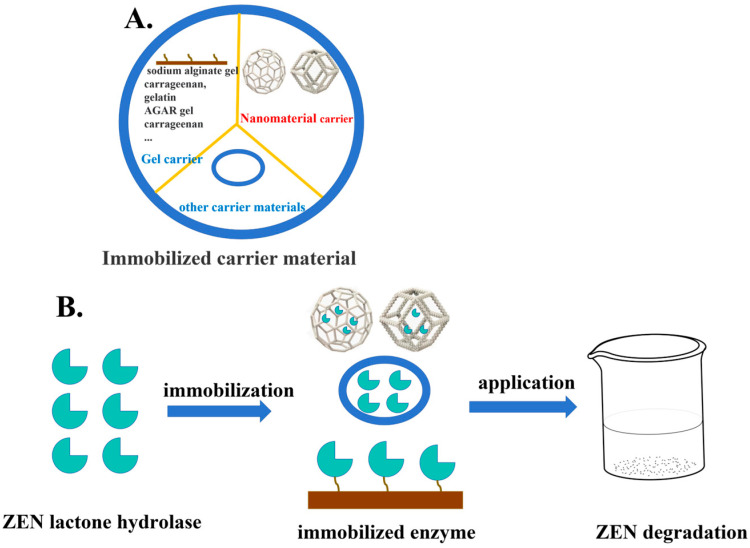
Immobilized enzymes degrade ZEN. (**A**) Immobilized carrier material. (**B**) The degradation of ZEN by lactonase immobilized via carrier-based assembly technology.

Enzyme immobilization technology refers to the process of fixing enzyme molecules onto inert or insoluble carriers such as gels or nanomaterials through methods including adsorption, encapsulation, crosslinking, or covalent bonding, as shown in Figure 3 [84]. This technology establishes stable covalent bonds between functional groups (-SH, -COOH, -NH2, etc.) on the carrier surface and amino acid residues of enzyme molecules, effectively overcoming the limitations of free enzymes, such as easy inactivation and difficult recovery. Particularly noteworthy is that carrier materials with high specific surface areas and porous structures can provide abundant active sites for enzyme immobilization, thereby significantly improving the catalytic efficiency of immobilized enzymes [85]. This technical strategy not only achieves high operational stability of enzyme systems but also maintains excellent catalytic performance, offering an ideal solution for enzyme applications in fields like food detoxification.

#### 4.2.1. Application of Gel-Based Enzyme Immobilization Technology for ZEN Detoxification

Enzyme immobilization via gel entrapment within three-dimensional polymeric networks significantly enhances enzymatic stability and reusability. This technique’s efficacy primarily depends on the judicious selection of gel matrices—including natural polymers (e.g., sodium alginate, carrageenan, gelatin, agar, and k-carrageenan) and synthetic alternatives (e.g., polyacrylamide, polyvinyl alcohol, and photo-crosslinkable resins)—coupled with optimization of immobilization parameters to maintain optimal activity-stability balance. In a representative study, Wang et al. [86] employed emulsion-gelation to microencapsulate ZEN-degrading ZLHY6 enzyme using sodium alginate matrix. The immobilized enzyme exhibited moderately shifted optimal conditions (35 °C, pH 8.0) compared to its free counterpart. Notably, it demonstrated remarkable thermal stability (retaining full activity after 4 h at 50 °C) and pH tolerance (80% residual activity after 2 h at pH 5.0). Simulated porcine gastrointestinal tests further confirmed enhanced robustness, with 61% activity retention following sequential 4 h gastric and 2.5 h intestinal digestion phases. Zheng et al. [87] developed an innovative montmorillonite-modified calcium alginate microsphere system for immobilizing ZLHY6 enzyme, successfully creating an eco-friendly and highly efficient SA/Mt./EZ microsphere for ZEN removal. The covalent immobilization of ZLHY6 onto the microspheres demonstrated superior ZEN removal efficiency compared to conventional entrapment and adsorption/cross-linking methods. The incorporation of montmorillonite (Mt) significantly enhanced the microspheres’ structural stability. Experimental results showed that 0.4 g of SA/Mt./EZ microspheres achieved 93.18% ZEN removal efficiency within 60 min when applied to 10 mL of ZEN-contaminated solution. When implemented in corn wet-milling processing, the SA/Mt./EZ microspheres effectively removed 63.02% and 60.40% of ZEN from corn gluten meal compared to untreated raw corn samples. These findings demonstrate that the SA/Mt./EZ microspheres represent a promising innovative material with significant potential for efficient ZEN detoxification in corn processing byproducts.

Bi [88] selected four immobilized carriers to immobilize ZENC enzyme, with each carrier carrying different activating groups to covalently bind with the amino terminus or ε-NH2 of the enzyme, thereby achieving enzyme immobilization on the carriers. The results showed that activated NH2-agarose beads exhibited the highest immobilization rate, reaching 86% in a 0.9 mg/L enzyme solution, significantly higher than the other three carriers. The specific activity of the immobilized enzyme was 49%. Moreover, activated NH_2_-agarose beads could endow ZENC enzyme with new characteristics, namely enhanced acid resistance and thermal stability.

Fu et al. [89] immobilized the purified zearalenone-degrading enzyme (rdZHD) using crosslinked poly (γ-glutamic acid)/gelatin hydrogel (CPE), followed by comparative analysis of thermal and pH stability between the free enzyme and its immobilized form (CPE-rdZHD). The immobilized enzyme exhibited significantly enhanced stability, retaining 53.4% activity after 1 h incubation at 50 °C. When exposed to higher temperatures (60 °C and 70 °C) for 45 min, CPE-rdZHD maintained 45.2% and 23.2% residual activity, respectively, while the free enzyme was nearly completely inactivated under identical conditions. In acidic conditions (pH 5.0), the immobilized form preserved 76.8% activity after 1 h, markedly higher than the free enzyme’s 15.6% retention. Similarly, under alkaline treatment (pH 9.0), CPE-rdZHD retained 33.1% activity after 15 min, whereas the free enzyme lost all catalytic function. This substantial stability improvement is primarily attributed to the protective microenvironment created by the CPE matrix, which effectively minimizes temperature and pH-induced hydrogen bond disruption and conformational changes in the enzyme structure.

Gel immobilization technology has significantly enhanced the thermal stability, pH tolerance, and operational stability of zearalenone lactonase (including ZLHY6, ZENC, and rdZHD), while improving its activity retention rate in complex environments. This provides a green and reusable enzyme immobilization solution for efficient ZEN removal.

#### 4.2.2. Applications of Nano-Immobilized Enzyme Technology for ZEN Detoxification

Recent advances in nanoscience and nanotechnology have provided innovative solutions for developing enzyme immobilization carriers. By incorporating enzymes into nanostructured materials, various nano-enzymes can be fabricated, including nanoporous particles, nanofibers, nanoflowers, nanogels, nanomembranes, metal–organic frameworks (MOFs), multi-walled or single-walled carbon nanotubes, as well as tunable-shape and size nanoparticles. The unique characteristics of nanomaterials—such as specific surface area, pore volume, chemical composition, surface charge, and conductivity—synergistically interact with the surface charge distribution, hydrophobicity, and amino acid composition of enzyme molecules. This interaction not only optimizes the preparation process and catalytic performance of nano-enzymes but also confers significant advantages, including reduced diffusion resistance, large specific surface area, and enhanced mass transfer efficiency [90,91,92].

Zhou et al. [93] successfully developed a sorting intron-functionalized hybrid nanoflower system (zearalenone lactase-inorganic hybrid nanoflowers, InHNF-ZHD518) through a biomineralization approach for rapid directional enzyme immobilization. This innovative system significantly enhanced both the activity and stability of zearalenone hydrolase ZHD518 while demonstrating superior degradation efficiency in complex food matrices. The immobilized enzyme exhibited remarkable advantages over its free counterpart, including 40–60% higher specific activity due to the increased enzyme-ZEN contact sites enabled by the nanoflower’s porous structure, maintained catalytic activity under extreme pH conditions (3–11) where free enzymes would denature, and excellent operational stability with over 70% activity retention after 8 consecutive catalytic cycles. In practical applications using beer samples, the InHNF-ZHD518 system degraded more than 50% of ZEN despite matrix interference that completely inactivated free enzymes, demonstrating its robust performance in complex food systems. This technology establishes a novel green immobilization strategy that bypasses the tedious purification steps required by conventional methods while simultaneously enhancing enzymatic stability and catalytic performance through organic-inorganic hybridization, showing tremendous potential for applications in food safety and environmental remediation [94]. Dong et al. [95] successfully constructed a tannic acid (TA)-based immobilized coating system on nylon membranes through systematic screening of three silane coupling agents. The results demonstrated that compared with the TA/3-glycidyloxypropyltrimethoxysilane (KH-560) system, the coatings formed by TA/3-aminopropyltriethoxysilane (APTES) and TA/3-(2-aminoethylamino) propyltriethoxysilane (AEAPTES) exhibited superior performance in glucose oxidase immobilization. The higher enzyme loading capacity and activity were attributed to a significant increase in nanoparticle structures, which provided more abundant active sites for enzyme immobilization. Notably, the optimized TA/AEAPTES/tetraethyl orthosilicate (TEOS)-Fe^3+^ immobilized ZHD not only displayed remarkable operational stability but also achieved catalytic activity surpassing that of the free enzyme. This important finding confirms that this biomimetic coating technology can serve as an efficient and stable multifunctional platform, offering a green and high-performance solution for industrial enzyme immobilization applications.

#### 4.2.3. Applications of Other Carrier Materials Technology for ZEN Detoxification

He et al. [96] developed a novel high-efficiency detoxification agent by immobilizing zearalenone-degrading enzyme (ZDE) derived from Aspergillus niger FS10 on rice husk (RH) carriers. This detoxification agent achieves efficient ZEN removal through the synergistic effects of physical adsorption by rice husk and biodegradation by the immobilized enzyme. The results demonstrated that the immobilized ZDE exhibited exceptional stability and catalytic activity: maintaining 70% enzymatic activity at 90 °C high temperature conditions, with activity retention rates of 90% after 30 days storage at 4 °C and 70% at 25 °C. Particularly noteworthy, the immobilized enzyme showed significantly superior ZEN removal efficiency compared to conventional commercial detoxifiers in simulated porcine gastrointestinal environments, achieving removal rates of 75.45% in artificial gastric fluid and 90.43% in intestinal fluid. Zhao [97] employed macroporous resin carriers to immobilize ZHD (IZHD) for corn oil detoxification. After immobilization, the enzyme showed increased Km and decreased Vm compared to its free form. Under optimal reaction conditions at 45 °C and pH 8, the ZEN content in corn oil was reduced to 533.28 μg/kg with a degradation rate of 92.46%. This technology offers multiple advantages, including wide availability of raw materials, low cost, and environmental friendliness, providing an economically efficient and sustainable solution for addressing ZEN contamination problems.

### 4.3. Current Applications and Cost Analysis of Enzymatic Detoxification Technologies

Although enzymatic detoxification processes offer distinct advantages such as high specificity and minimal secondary pollution, their economic viability has often been questioned. Cost–benefit analyses of enzyme applications in industrial settings demonstrate that their economic performance primarily depends on key factors including enzyme type, production processes, and scale effects. While the initial investment cost for enzymatic treatment is typically 2–5 times higher than conventional methods, its significant advantages—including improved catalytic efficiency, reduced energy consumption, and environmental friendliness—confer clear competitive advantages in long-term operations [85]. Currently, innovative strategies such as immobilized enzyme technology (enabling 5–20 reuse cycles with over 60% cost reduction) [84], AI-assisted enzyme design, and scaled-up production are continuously optimizing the overall cost-effectiveness of enzyme preparations. With the rapid development of computer-aided design and bioinformatics technologies, the production efficiency of enzyme preparations is expected to further improve [98]. Although specific data remain limited due to commercial confidentiality, ongoing technological advancements and growing market demand will undoubtedly drive continuous improvement in the economic feasibility of enzymatic processes.

## 5. Conclusions and Future Perspectives

Research on zearalenone lactone hydrolases has established an efficient and environmentally sustainable solution for mycotoxin contamination management. Through in-depth exploration of its catalytic mechanism and structural characteristics, researchers have confirmed that the enzyme can specifically hydrolyze the lactone ring of ZEN, significantly reduce its estrogen activity, and show important application value in the fields of feed detoxification, food processing, and environmental restoration. Advances in molecular engineering techniques—including rational design, directed evolution, and computational optimization—have further improved the enzyme’s acid tolerance, thermal stability, and catalytic efficiency, enhancing its adaptability to complex practical environments. Immobilization technologies have addressed the limitations of free enzymes regarding instability and difficult recovery, laying the foundation for large-scale applications. However, in the application of enzyme stability, it is usually impossible to ensure the balance between enzyme stability activities, and the introduction of new mutations will weaken other functions, resulting in mutual restriction between stability and catalytic activity. Sacrificing the catalytic activity to improve the thermal stability cannot give full play to the utilization value of the enzyme, and researchers are focused on assisting the co-evolution of the thermal stability and activity of the enzyme through combined strategies so as to inject more feasibility into improving the thermal stability and catalytic activity of the enzyme in the future. It is also necessary to focus on the development of an efficient expression system, the innovation of intelligent immobilized materials, and the verification of degradation efficiency in practical environmental applications, and to explore the synergistic effect with other toxin-degrading enzymes in order to achieve wider application.

## Figures and Tables

**Table 1:** Enzymes degrading from various microbial sources.

Source Microorganism	Gene Name	Substrate	Expression Host	Optimum pH	Optimum Temperature/°C	Enzyme Activity Value
*Clonostachys rosea IFO 7063*	ZHD101	ZEN	*S. pombe and E. coli*	9.0–10.0	37–45	NR [30]
*Cladophialophora bantiana*	CLA	ZEN	*E. coli* BL21	7.0	40	114.8 U/mg ^a^ [38]
*Exophiala aquamarina*	EXO	ZEN	*E. coli* BL21	9.0	40	459.0 U/mg ^a^ [38]
*Trichoderma aggressivum*	TRI	ZEN	*E. coli* BL21	9.5	40	239.8 U/mg ^a^ [38]
*Gliocladium* spp.	ZHDR52, ZHDP83	ZEN, α/β-ZEL α/β-ZAL	*E. coli* BL21 (DE3)	9.0	45	ZEN 196.11 U/mg ^a^, 229.64 U/mg ^a^ >90% removal of α/β-ZEL and α/β-ZAL in 6 h [39]
*Exophiala aquamarina CBS 119918*	ZHDAY3	ZEN, α/β-ZEL α/β-ZAL	*E. coli* BL21 (DE3)	9.5	40	ZEN 157.5 U/mg ^a^ α-ZEL 79.6 U/mg ^a^ α-ZAL 115 U/mg ^a^ β-ZEL 71.7 U/mg ^a^ β-ZAL 53.0 U/mg ^a^ [29]
*Aeromicrobium strain*	ZenH	ZEN	*E. coli*	7.0	55	The specific activity of ZenH against ZEN is 7.05 U/mg ^a^ [40]
*Trichoderma aggressivum*	ZHD-P	ZEN	*E. coli* BL21 (DE3)	7.5–9.0	45	191.94 U/mg ^a^ [41]
*Clonostachys rosea strain GrZ7*	PR-ZHD	ZEN	*E. coli* Rosetta TM (DE3)	8.5	30	Complete degradation [42]
*Fonsecaea multimorphosa CBS 102226*	ZHD11C	ZEN, α/β-ZEL α/β-ZAL	*E. coli* DH5α and BL21 (DE3)	NR	45	ZEN 55.8 U/mg ^a^ α-ZEL 21.6 U/mg ^a^ α-ZAL 37.3 U/mg ^a^ β-ZEL 9.3 U/mg ^a^ β-ZAL 6.3 U/mg ^a^ [43]
*Rhodococcus erythropolis PFAD 8-1*	ZENA	ZEN	NR	8.2	50–60	NR [44]
*Phialophora attae*	ZHD11F	ZEN	*E. coli* BL21 (DE3)	8.0	35	40.04 U/mg ^a^ [45]
*Monosporascus* sp. *GIB2.*	ZENM	ZEN, α/β-ZEL, α-ZAL	*E. coli* BL21 (DE3) and *E. coli* DH5α	9.0	60	ZEN 333 U/mg ^a^ α-ZEL 316 U/mg ^a^ α-ZAL 300 U/mg ^a^ β-ZEL 210 U/mg ^a^ [37]
*Gliocladium roseum*	S162P/S220R	ZEN	*E. coli* JM109 and *E. coli* BL21 (DE3)	7.0	35	371 U/mg ^a^ 224 U/mg ^a^ [46]
*Rhinocladiella mackenziei*	ZHD518	ZEN, α/β-ZEL α/β-ZAL	*E. coli* BL21 (DE3)	8.0	40	ZEN 207.0 U/mg ^a^ α-ZEL 23.0 U/mg ^a^ α-ZAL 119.8 U/mg ^a^ β-ZEL 64.7 U/mg ^a^ β-ZAL 66.5 U/mg ^a^ [47]
*Rhinocladiella mackenziei CBS 650.93*	RmZHD	ZEN	*E. coli* BL21 (DE3)	8.6	45	1.27 U/mg ^c^ [48]
*Exophiala spinifera CBS 89,968*	ZHD_LD	ZEN	*E. coli*	9.0	50	1.15 ± 0.04 U/mg ^c^ [49]
*Glocladium roseum*	ZENG	ZEN, α-ZEL α-ZAL	*E. coli* BL21 (DE3)	7.0	38	ZEN 315.0 U/mg ^a^ α-ZEL 187 U/mg ^a^ α-ZAL 117 U/mg ^a^ [50]
*Glodophialophora bantiana*	CbZHD	ZEN	*E. coli* BL21 (DE3)	8.0	35	0.688 U/mg ^c^ [51]
*Marasonina brannaea*	mbZHD	ZEN	*E. coli* BL21 (DE3)	NR	NR	200 U/mg ^d^ [52]
*Neurospora crassa*	ZENC	ZEN	*P. pastoris*	8.0	45	530.4 U/mg ^a^ [36]
*Glocladium roseum*	ZHD101	ZEN, α/β-ZEL	*P. pastoris* GS115	9.5	37	ZEN 4976.5 U/mg α-ZEL1257.1 U/mg β-ZEL 780.9 U/mL (15 min) [53]
*Phialophora americana*	ZHD607	ZEN	*P. pastoris*	8.0	35	4940 U/mg ^b^ [35]
*Bacillus subtilis YT-4*	ZENY	ZEN	*E. coli* DH5α and *P. pastoris* GS115	8.0	37	60% degradation in 6 h. 95% degradation in 36 h [54]
*Glocladium roseum*	zlhy-6	ZEN	*P. pastoris* GS115	NR	NR	10.0 U/mL ^a^ [55]

Note: ^a^: The amount of enzyme required to degrade 1 μg of Zen per minute. ^b^: The amount of enzyme required to degrade 1 μg of Zen per hour; ^c^: The amount of enzyme required to degrade 1 umol of ZEN per minute; ^d^: every minute quantity of enzyme required for the degradation of 1 ng ZEN. NR, not reported.

**Table 2:** Molecular modification and optimization effects of lactone hydrolases under different strategies.

Strain Name	Enzyme	Transformed Strategy	Optimization Method	Active Effect
*Clonostachys rosea*	ZHD101	Directed evolution	Combination mutation (Screen out the optimal mutant V153H-V158F, zhd101.1)	Its specific activity is 1.1 times higher than that of the wild type, and it has better thermal stability and pH stability [63]
*Clonostachys rosea*	ZHD	Directed evolution	site-directed mutagenesis	The pKa values of the residues E126 and H242 catalyzed by ZHD enzyme were reduced [64]
*Trichoderma aggressivum*	ZHD-P	Directed evolution	The *E. coli* cell surface display system of ZHD-P and gene screening were constructed	Intracellular ZHD-P remained 100% active after incubation at 25–40 °C for 1 h. The surface displayed ZHD-P showed high activity against ZEN and remained 80% active after incubation at pH 5.0–11.0 for 12 h [41]
*Clonostachys rosea*	ZHD101	Rational design	site-directed mutagenesis (There are 9 mutants, represented by S220R and S220W.)	The thermal melting temperature (Tm) of the 9 mutants increased by 0.4–5.6 °C. Among them, S220RandS220W showed the best thermal stability, with Tm increasing by 5.6 °Cand 4.0 °C, respectively. The thermal half-inactivation time at 45 °C was extended by 15.4 times and3.1 times, respectively. The relative enzyme activities were 70.6% and 57.3% of those of the wild type, respectively [65]
*Gliocladium roseum*	ZHD101	Rational design	mutation	Under the conditions of pH 4.2 and 37 °C, the degrading enzyme activity of 8 mutant sites increased from 7.69 U/mg to 38.67 U/mg. After evolution, Km decreased from 283.61 μM to 75.33 μM [66]
*Clonostachys rosea*	ZHD101	Rational design	site-directed mutagenesis (V153H)	The activity of V153H against ZEN remained unchanged, but its specific activity against α-ZOL increased by 3.7 times, the affinity for substrates decreased by 2.7 times, but the conversion rate increased by 5.2 times [67]
*Gliocladium roseum*	ZENG	Rational design -structure -based modification.	site-directed mutagenesis (H134F/S136F, H134I/S13 6I, H134L/S136L)	The three mutants retained 40% of their activity after being incubated at 48 °C for more than 7 min [50]
*Phialophora macrospora*	ZHD11A	Rational design	site-directed mutagenesis (I160Y-G242S)	The mutant can still maintain about 40% residual activity at 55 °C for 10 min. Compared with ZHD11A, the specific activity of I160Y-G242S is also increased by 2 times, from 220 U/mg to 450 U/mg [68]
*Fonsecae monophora*	Zhd11B	Rational design -structure -based modification.	site-directed mutagenesis (T158H)	The relative activity of the T158H mutant against α-ZOL increased by 1.3 times [69]
*Phialophora americana*	ZHD607	Rational design	site-directed mutagenesis (ZHDM1 and I160Y)	The degradation activity of the mutant ZHDM1 and I160Y for ZEN was 2.9 times and3.4 times that of ZHD607, respectively [35]
*Rhinocladiella mackenziei*	Zhd518	Rational design	site-directed mutagenesis (N156H)	The degradation efficiency of α-ZOL is 3.3 times that of the wild type [47]
*Rhinocladiella mackenziei*	RmZHD	Rational design	site-directed mutagenesis (V153H and Y160A)	The degradation efficiency of V153H for α-ZOL has increased by 3.17 times, and the hydrolysis activity of Y160A for α-ZOL has increased by 70% [70]
*Rhodococcus erythropolis PFA D8-1*	ZENA	Rational design	site-directed mutagenesis (D264A, D264L, D264N)	The catalytic triplet was identified and defined as Ser-128-His303-Asp-153, and it had degradation activity [44]
*Monosporascus* sp. *GIB2*	ZENM	Rational design	site-directed mutagenesis (G163S)	The catalytic activity of the mutant against α-ZOL (kcat/Km 0.223 min^−1^ μM^−1^) is higher than that against ZEN (kcat/Km 0.191 min^−1^ μM^−1^), and α-ZOL is its optimal substrate. The mutant can change the substrate specificity of lactone hydrolase [37]
*Bacillus subtilis YT-4*	ZENY	Rational design	Site-directed mutagenesis (N∆11 and N5V)	The first 11 amino acids at the N-terminal were replaced by the first 13 amino acids in the N-terminal region of ZHD11C, especially the fifth residue n was replaced by V, and a 25% stability improvement was achieved at 45 °C [54]
*Clonostachys rosea*	ZHD101	Rational design	introduce disulfide bonds (D143C/P18 1C)	The residual activity after treatment at 50 °C for 2 min is approximately twice that of the wild type [71]
*Fonsecaea multimorphosa CBS 102226*	ZHD11C	Rational design	gene selection	After incubation at 45 °C for 1 h, it retained nearly 90% of its activity. After incubation at pH6.5 to 9.0 for 12 h, it still retained over 12% of its activity and was capable of hydrolyzing α-ZAL, α-ZOL, β-ZAL, and β-ZOL [43]
*Fonsecae monophora*	Zhd11B	Rational design -structure -based modification	Hat domain swap (V131-L172 replaces the corresponding area of Zhd518)	The activities against ZEN and α-ZAL and β-ZAL were increased by 1.5, 1.6 and 2.9 times, respectively [69]
*Clonostachys rosea*	ZHD101	Computer-aided rational design-Computational design of pH-activity profiles for enzymes	site-directed mutagenesis (D157K and E171K)	M2 (D157K) and M9 (E171K) moderately enhanced the catalytic efficiency of ZHD101 under acidic conditions. The kcat/K_m_ of the two mutants was 1.34 and 2.06 times that of the wild type, respectively [31]
*Exophiala aquamarina CBS 119918*	ZHDAY3	Computer-aided rational design	site-directed mutagenesis (N153H)	The hydrolytic activity increased from 115.1 ± 2.1 U/mg to 253.3 ± 4.3 U/mg [29]
*Clonostachys rosea*	ZHD101	Computer-aided rational design-Virtual saturation mutation based on flexible regions	site-directed mutagenesis (N156F, S194T and T259F)	The enzyme activities of the three mutants were 95.8%, 131.6% and 169.0%, respectively, compared with the wild type. The Tm of the double mutant TIN156F/S194T and the triple mutant N156F/S194T/T259F increased by 6.7 °C and 6.1 °C, respectively [72]
*Gliocladium roseum*	ZENG	Computer-aided rational design-MD simulations	site-directed mutagenesis (S162P/S220R)	S162P/S220R mutant under 55 °C half-life (t1/2) is 36.8 times higher than wild-type enzyme, Tm significantly increased by 8.2 °C [46]
*Glocladium roseum*	ZLHY6	Computer-aided rational design -Computer virtual saturation mutation	site-directed mutagenesis (H134 W)	After heat treatment at 45 °C for 20 min, the relative enzymatic activity of mutant H134W was 10 times that of the wild type, and it retained a certain activity against ZEN (41.33%) [73]
*Glocladium roseum*	ZLHY6	Computer-aided rational design -Computer virtual saturation mutation	introduce disulfide bonds (Q45C/A253 C)	After heat treatment at 45 °C for 20 min, the relative enzymatic activities of Q45C/A253C were 3.1 times that of the wild type, respectively, and they retained a certain activity (41.33%) against ZEN [73]
*Rosellinia necatrix*	ZHRnZ	Computer-aided rational design	site-directed mutagenesis (E122R)	The catalytic efficiency of E122R is 1.3 times higher than that of the wild type [74]

## Data Availability

No new data were created or analyzed in this study. Data sharing is not applicable to this article.
